# HLA-Haplotypes Influence Microbiota Structure in Northwestern Mexican Schoolchildren Predisposed for Celiac Disease or Type 1 Diabetes

**DOI:** 10.3390/microorganisms11061412

**Published:** 2023-05-27

**Authors:** Sandra V. Aguayo-Patrón, Omar A. Trujillo-Rivera, Fernanda Cornejo-Granados, Adrian Ochoa-Leyva, Ana M. Calderón de la Barca

**Affiliations:** 1 Coordinación de Nutrición, Centro de Investigación en Alimentación y Desarrollo A.C., Hermosillo 83304, Mexico; sandra.aguayo@ciad.mx (S.V.A.-P.); otrujillo121@estudiantes.ciad.mx (O.A.T.-R.); 2 Departamento de Microbiología Molecular, Instituto de Biotecnología, Universidad Nacional Autónoma de Mexico, Cuernavaca 62210, Mexico; fernanda.cornejo@ibt.unam.mx (F.C.-G.); adrian.ochoa@ibt.unam.mx (A.O.-L.)

**Keywords:** HLA DQ2 and DQ8, celiac disease, type 1 diabetes, microbiota, schoolchildren, Northwestern Mexico

## Abstract

To contribute to and elucidate the participation of microbiota in celiac disease (CD) and type 1 diabetes (T1D) development, we evaluated the influence of HLA haplotypes, familial risk, and diet on the microbiota of schoolchildren. We conducted a cross-sectional study on 821 apparently healthy schoolchildren, genotyping HLA DQ2/DQ8, and registering familial risk. We analyzed the fecal microbiota using 16S rRNA gene sequencing, and autoantibodies for CD or T1D by ELISA. After analyses, we created three groups: at-high-risk children (Group 1), at-high-risk children plus autoantibodies (Group 2), and nonrisk children (Group 3). HLA influenced the microbiota of Groups 1 and 2, decreasing phylogenetic diversity in comparison to Group 3. The relative abundance of Oscillospiraceae UCG_002, *Parabacteroides*, *Akkermansia*, and *Alistipes* was higher in Group 3 compared to Groups 1 and 2. Moreover, Oscillospiraceae UCG_002 and *Parabacteroides* were protectors of the autoantibodies’ positivity (RRR = 0.441 and RRR = 0.034, respectively). Conversely, *Agathobacter* was higher in Group 2, and Lachnospiraceae was in both Groups 1 and 2. Lachnospiraceae correlated positively with the sucrose degradation pathway, while the principal genera in Group 3 were associated with amino acid biosynthesis pathways. In summary, HLA and familial risk influence microbiota composition and functionality in children predisposed to CD or T1D, increasing their autoimmunity risk.

## 1. Introduction

CD and T1D are autoimmune diseases with common risk factors. They share the genetic predisposition given by the human leukocyte antigen (HLA-DQ and DR) genes as well as several environmental risk factors, such as infections, antibiotic usage, breastfeeding, and diet. HLA molecules, which are encoded in the high-risk alleles of the major histocompatibility complex, bind gluten peptides or islet autoantigens and present them to reactive T-cells, starting the autoimmunity response. Although HLA molecules are indispensable, they are not sufficient for autoimmunity to develop [1].

Interestingly, most of the environmental factors related to CD and T1D development have an impact on the gut microbiota. For example, diet is a well-known modulator of the microbiota, and it has been described as a risk factor for autoimmunity [2,3]. Moreover, intestinal parasites influence gut microbiota composition in children with CD or T1D autoimmunity [4].

The microbiota profile is altered in patients with CD or T1D compared to healthy controls, mainly represented by an altered Firmicutes/Bacteroidetes ratio and an increased abundance of Bacteroides among other genera [5,6]. These variations in microbiota structure also affect its functionality related to human metabolism. For instance, bacterial abundance related to amino acid metabolism and other functional pathways increased before the onset of CD [7]. There have also been increases in carbon, sugar, and iron metabolism associated with changes in the microbiota and their metabolites at the onset of T1D [8].

Longitudinal studies following at-risk children from birth to disease diagnosis contribute to identifying microbiota structure and functional changes that lead to CD or T1D development [7,9]. However, such studies present problems to follow, and high costs make them unfeasible in some contexts. For instance, in Mexico, more than 20% of the population is not affiliated with public health services [10], and the migration of farmworkers from the poorest areas to more developed ones exacerbates the problem [11], making participant enrollment difficult and leading to significant losses in follow-up. Therefore, we propose a cross-sectional approach to identify at-risk children for CD or T1D and assess the presence of autoantibodies and their diet at public elementary schools. Then, our aim was to evaluate the influence of HLA-DQ2/DQ8, familiar autoimmunity risk, and diet on the microbiota structure and functionality of apparently healthy schoolchildren.

## 2. Materials and Methods

We conducted a cross-sectional study from 2015 to 2017 in Hermosillo, located in northwest Mexico. The study protocol was reviewed and approved by the Institutional Ethics Committee (CE/016/2014). Participants were schoolchildren between 7 and 12 years old; therefore, informed consent was obtained from their parents or legal guardians. Data collection was carried out at the facilities of elementary schools in semimarginalized urban areas, with prior authorization from the principals and teachers. We excluded from the study children who had taken antibiotics in the last month or had undergone any medical treatment that could modify the intestinal microbiota composition.

### 2.1. Genotyping and Risk for CD and T1D Identification

Dried blood spots were taken from schoolchildren for HLA-DQ2 and DQ8 analysis, as previously done [12]. Genetic risk for CD and T1D was classified according to the risk gradient we proposed for the Northwestern Mexico population [13].

Children with high genetic risk were identified, and their mothers or legal guardians were interviewed to obtain their familiar and clinical histories. The interview focused on personal and family variables related to disorders, such as whether the family members had autoimmune diseases, type of birth, lactation regime, complementary feeding, and antibiotic usage in the first year of life [1,14]. Considered risk factors were first-degree relatives with CD, T1D, or other autoimmune disease, c-section birth, breastfeeding < 6 mo., age of solid food introduction < 6 mo., and a high number of antibiotic cycles/year. Children that presented at least 3 risk factors were asked to provide a blood sample for antibody analysis.

### 2.2. Autoantibody Detection

A peripheral blood sample was collected from children with a high risk for CD or T1D, and serum antibodies and autoantibodies were analyzed by ELISA (enzyme-linked immunosorbent assay). IgA and IgG anti-gliadins and anti-transglutaminase antibodies (for CD), as well as anti-insulin antibodies (for T1D), were evaluated following the general procedure previously done in our lab [15]. Additionally, for T1D, anti-tyrosine phosphatase (IA2) and anti-glutamate dehydrogenase (GADA) antibodies were quantified with the GAD/IA-2 Antibody Screen ELISA Test Kit from KRONUS (Kronus, ID, USA).

After autoantibodies’ detection, two groups were formed: Group 1: children with high-risk HLA, negative for autoantibodies; and Group 2: children with high-risk HLA, positive for autoantibodies. Additionally, Group 3 was a negative reference group composed of children without a genetic risk for CD or T1D. Children in Groups 1 and 3 were matched in sex and age to those in Group 2.

### 2.3. Microbiota and Diet Analysis

Feces samples from all the schoolchildren included in the three study groups were collected and transported on ice to the lab. Each sample was homogenized, and an aliquot was stored at −60 °C for DNA extraction. DNA extraction was performed with the QIAmp Fast DNA Stool Mini Kit (QIAGEN, Hilden, Germany) following the manufacturer’s protocol. Quantity and quality were evaluated in a spectrophotometer Nanodrop 2000 (Thermo Scientific, Pittsburg, PA, USA). For microbiota analysis, the V3-V4 region of the 16S rRNA gene was amplified with primers S-D-Bact-0341-b-S-17 (5′-CCTACGGGNGGCWGCAG-3′) and S-D-Bact-0785-a-A-21(5′-GACTACHVGGGTATCTAATCC-3′) [16]. After amplification, amplicons were checked in 2% agarose gel, purified with Ampure XP beads (Beckman Coulter, Inc., Brea, CA, USA), and barcoded according to the 16S Metagenomics Sequencing Library Preparation user’s guide from Illumina. All libraries were mixed in equal concentrations and sequenced on the Illumina Miseq (San Diego, CA, USA) sequencing platform using a 2 × 150 paired-end format at the National Institute of Genomic Medicine (INMEGEN) in Mexico City, México.

The schoolchildren’s diet was evaluated with two or three non-consecutive 24 h recalls. Details of meal preparation were discussed with the mothers or caregivers when needed. Energy, macronutrients, and fiber consumption were analyzed as described by Ortega et al. [17].

### 2.4. Bioinformatics and Statistical Analysis

Descriptive analysis was made for participants’ characteristics and dietary data. For all the variables, comparisons were made with ANOVA when normality and equal variances were achieved; Aspin–Welch test was used when normal data but unequal variances were found; and Kruskal–Wallis test was employed for abnormal data, all using the software NCSS 2021.

Analysis of microbial diversity and taxonomy was made in QIIME2 [18]. Primers were removed from the imported sequences using the Cutadapt tool and denoised with DADA2. Sequences with Phred quality score < 20 were excluded. Faith’s PD, Pielou, and Shannon indices were estimated to analyze alpha diversity. Weighted UniFrac distances and principal coordinate analysis of Bray-Curtis dissimilarities were used to evaluate beta diversity.

Filtered sequences were clustered into operational taxonomic units (OTUs) with a 99% similarity threshold, and taxonomy was assigned using SILVA database. Relative abundances at phylum, family, and genus levels, were calculated in Excel and plotted in GraphPad 8.0. For comparisons between groups, only genus with abundance ≥ 1% were included. Covariable significance was tested, and adjustments were made, when necessary, with a GLM model in NCSS 2021. Multinomial logistic regression analysis of microbial abundance in relation to genetic risk and autoantibody presence was performed in STATA MP17.

Microbiota functional capacity was predicted based on the taxonomic composition using PICRUSt2 2021.11_0 [19]. KEGG pathways were mapped and classified with the BioCyc database. The main functional categories’ relative abundance was compared between groups, and the most abundant pathways in those categories were identified. The correlation between these pathways and microbiota markers at the genus level was calculated with Pearson’s coefficient in STATA MP17. A heat map for these correlations was plotted in OriginLab 2023.

## 3. Results

HLA-DQ2 and DQ8 genotyping were performed on 821 schoolchildren. Among them, 302 had haplotypes or allelic combinations predisposing them to T1D or CD. The parents of 57 of them decided not to follow the study. Therefore, clinical history data was collected from 245 children, and additional risk factors for T1D or CD were identified in 94 of them. Seven of the children declined to provide a blood sample; thus, autoantibodies were analyzed in 87 children. Among the tested children, 18 had positive results. Specifically, IAA was found in fourteen children, two were positive for IgA antitransglutaminase antibodies, one child had both IAA and anti-transglutaminase antibodies, and one child had IAA, GADA, and IA2 positivity, but not symptoms, according to the endocrinologist consulted.

Table 1 shows the characteristics and dietary data of the participants in the three study groups. No variable differed between groups.

### 3.1. Fecal Bacterial Microbiota

The fecal bacterial microbiota was analyzed in the three study groups. A total of 1,010,765 raw paired-end reads were obtained. After removing the primers and filtering by quality score, 805,610 reads were retained, with an average of 14,919 reads per sample. One sample from a child without genetic risk (Group 3) did not meet the quality filters and was removed from the analysis.

**Figure 1:**
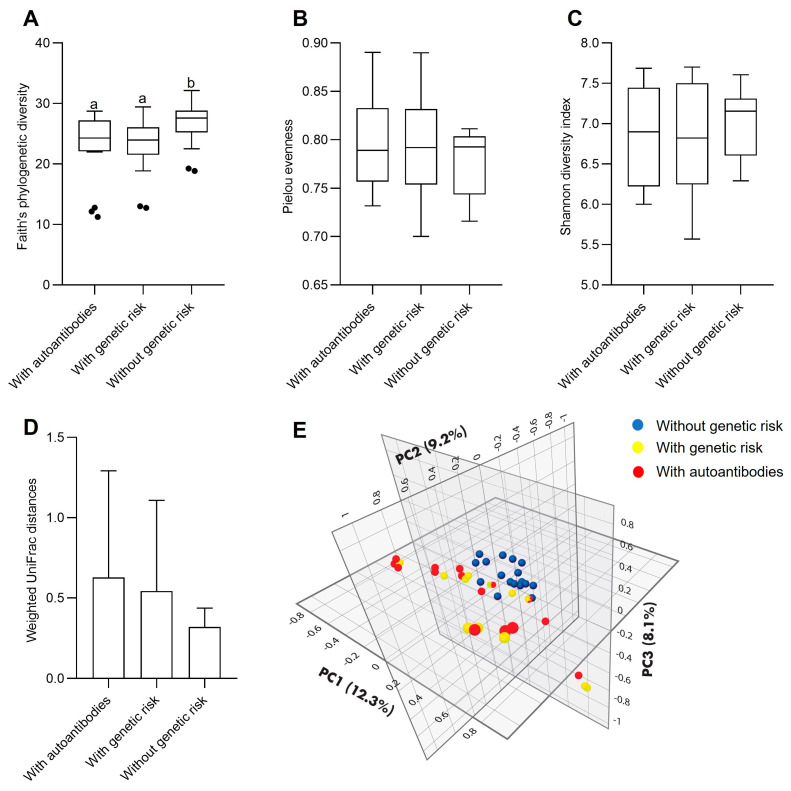
Microbiota diversity comparison between the study groups. (**A**–**C**) Alpha diversity comparison. (**A**) Faith’s phylogenetic diversity. (**B**) Pielou evenness. (**C**) Shannon diversity index. (**D**,**E**) Betha diversity. (**D**) Weighted UniFrac distances. (**E**) Principal coordinates analysis of Bray–Curtis dissimilarities. Group 1: children with genetic risk without autoantibodies. Group 2: children with genetic risk and autoantibodies. Group 3: children without genetic risk. Different letters indicate significant differences.

With respect to microbial ecology, phylogenetic diversity (Faith’s PD) was higher in Group 3 children compared to those in Group 1 (*p* = 0.017) and Group 2 (*p* = 0.025) (Figure 1A). However, the Pielou and Shannon indices did not differ between groups (Figure 1B,C). Beta diversity evaluated as weighted UniFrac distances was also not different between groups (Figure 1D). Principal component analysis of Bray-Curtis dissimilarities showed that samples from Group 3 tended to be homogeneously grouped; however, the high dispersion found in Groups 1 and 2 made them overlap (Figure 1E).

Taxonomic analysis revealed that the predominant phylum was Bacteroidetes, with a relative abundance of 55.7%, 55.4%, and 58.9% in Groups 1, 2, and 3, respectively. It was followed, in abundance order, by Firmicutes (Group 1: 29.9%; Group 2: 31.0%; and Group 3: 31.9%), Proteobacteria (Group 1: 8.8%; Group 2: 7.6%; and Group 3: 4.1%), Cyanobacteria (Group 1: 3.9; Group 2: 4.4%; and Group 3: 2.0%), Verrucomicrobia (Group 1: 1.1%; Group 2: 0.9%; Group 3: 2.1%), and Actinobacteria (Group 1: 0.6%; Group 2: 0.7%; Group 3: 1.0%). There were no significant differences between groups (*p* > 0.05). However, there was a trend towards a higher abundance of Verrucomicrobia (*p* = 0.061) and Actinobacteria (*p* = 0.056) in Group 3.

**Figure 2:**
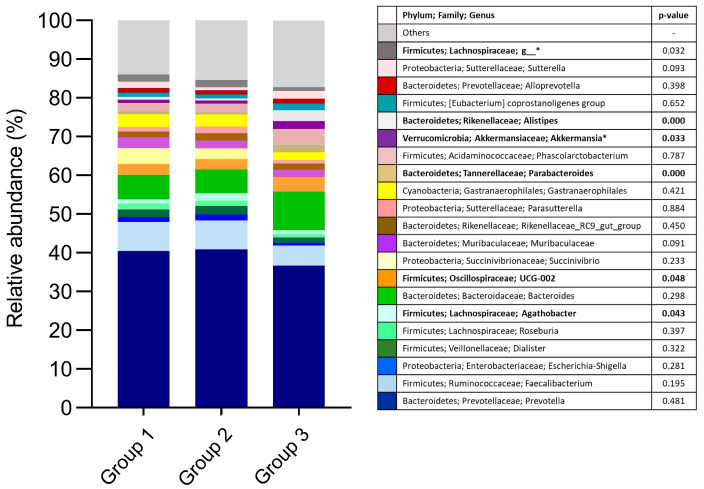
Relative abundances at genus level in the three study groups. Group 1: children with genetic risk without autoantibodies. Group 2: children with genetic risk and autoantibodies. Group 3: children without genetic risk. Bold letters mark genus differences between groups. * Significance found after adjusting.

Figure 2 shows the relative abundances at the family and genus levels in the three study groups. As can be seen, *Agathobacter* abundance was higher in Group 2 compared to Groups 1 and 3 (*p* = 0.043). Meanwhile, Lachnospiraceae was higher in Groups 1 and 2 than in Group 3 (*p* = 0.032). In contrast, Oscillospiraceae UCG-002 (*p* = 0.048), *Parabacteroides* (*p* = 0.000), *Akkermansia* (*p* = 0.033), and *Alistipes* (*p* = 0.000) were more abundant in Group 3 than in Groups 1 and 2.

Furthermore, according to multinomial logistic regression analysis, a higher relative abundance of *Parabacteroides* was associated with a significantly decreased risk of belonging to Group 2 (RRR = 0.034, *p* < 0.001). Similarly, a higher relative abundance of Oscillospiraceae UCG-002 was also found to be a protective factor, as it was associated with a decreased risk of being an individual of Group 2 (RRR = 0.441, *p* < 0.01).

### 3.2. Microbiota Functional Analysis

Analysis of microbiota functionality generated a total of 484 KEGG pathways that were assigned to 30 functional categories. Amino acid biosynthesis, nucleoside and nucleotide biosynthesis, cofactor, carrier, and vitamin biosynthesis, carbohydrate metabolism, and fatty acid and lipid biosynthesis accounted for more than 65% of the pathway abundance in the three study groups. Amino acid biosynthesis (17.2% vs. 16.0%; *p* = 0.024) and nucleoside and nucleotide biosynthesis (16.9% vs. 15.9%; *p* = 0.021) were more abundant in Group 3 than in Group 2, whereas cofactor, carrier, and vitamin biosynthesis were higher (13.2% vs. 12.1%; *p* = 0.032) in Group 2 than in Group 3. No differences were detected in carbohydrate metabolism or fatty acid and lipid biosynthesis among groups (*p* > 0.05).

**Figure 3:**
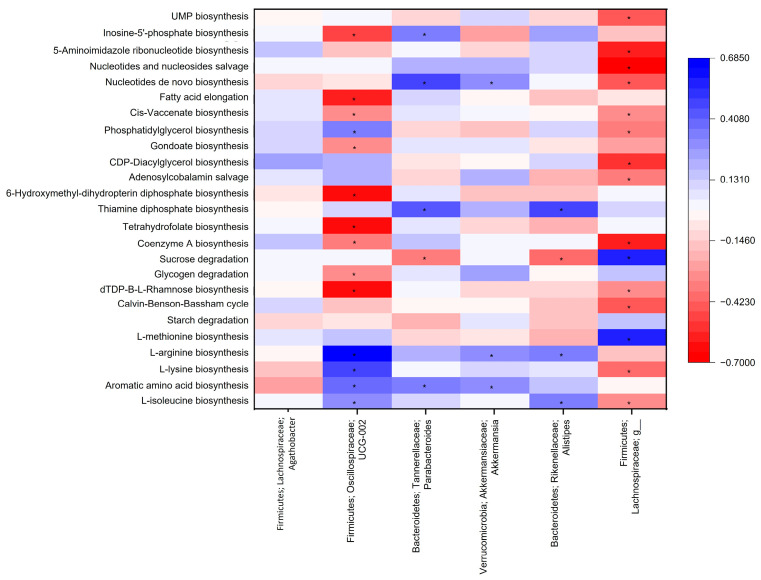
Heat map of the Pearson’s correlation index between differential genus between study groups and the most abundant metabolic pathways. * Statistical significance (*p* < 0.05).

A correlation analysis performed between microbiota genera with differences among groups and the most abundant pathways in the considered functional categories are shown in Figure 3. It is notable that the most abundant genera in Group 3 (Oscillospiraceae UCG-002, *Parabacteroides*, *Akkermansia*, and *Alistipes*) are positively and significantly correlated with L-isoleucine, aromatic amino acids, L-lysine, or L-arginine biosynthesis. Meanwhile, *Agathobacter* and Lachnospiraceae had no or negative correlations with these pathways, except for L-methionine biosynthesis and the last genus.

Among carbohydrate metabolism pathways, only sucrose degradation correlated positively with Lachnospiraceae. Thiamine diphosphate biosynthesis was associated with *Parabacteroides* and *Alistipes*, but no other genus correlated positively with cofactor, carrier, or vitamin biosynthesis pathways. Fatty acid and lipid biosynthesis pathways correlated negatively with Oscillospiraceae UCG-002, but phosphatidylglycerol biosynthesis correlated positively with it. In nucleoside and nucleotide biosynthesis, *Parabacteroides* correlated positively with both inosine-5’-phosphate and nucleotide de novo biosynthesis, while *Akkermansia* correlated positively only with nucleoside and nucleotide biosynthesis.

## 4. Discussion

Our goal was accomplished. We were able to evaluate the influence of familiar and genetic risk factors for T1D or CD and diet on the microbiota structure and functionality of apparently healthy schoolchildren from a semimarginalized urban area in Northwest Mexico. An important proportion of the 821 children presented at least one risk allele for DT1 or CD, and it was filtered by familiar risk and clinical history related by mothers. Then, autoantibody analyses showed that 2% of the children were positive at least for anti-insulin antibodies. Therefore, we created Groups 1, 2, and 3 for genetic risk, positive autoantibodies, and negative autoantibodies for genetic risk children, respectively.

In general, there were no differential effects of diet among the three groups on the microbiota structure, with the predominance of the Bacteroidetes phylum (55–59%), followed by Firmicutes (30–32%), and ending with only 0.6–1% Actinobacteria. The observed proportions were similar to the ones previously found in our previous studies in the same population [4,6]. Similar to the phyla results, alpha diversity was not different among the three children groups. However, phylogenetic diversity (Faith PD) was higher in Group 3 than in Groups 1 and 2. Although only 4/18 of our children in Group 2 could be considered at the onset of T1D or CD, our results coincide with those by Leonard et al. [7] for the alpha diversity of children at CD onset. Kostic et al. [9] found differences in alpha diversity after comparing high-risk children for DT1 who seroconverted (as our Group 2) with no seroconverted ones (as our Group 1 children).

A greater phylogenetic diversity is associated with microbiota stability and resilience, as well as with the ability to perform complex metabolic functions. Loss of microbiota diversity and gut dysbiosis have been related to a westernized lifestyle and several non-communicable and autoimmune diseases [20]. Moreover, as seen in our analysis of Bray-Curtis dissimilarities, although not significantly different, Group 3 children tended to lump together more homogeneously. This is due to the similarity in the taxa of their microbiota, suggesting high stability [21].

All children in the studied groups were similar in sex, age, weight, height, and diet. Thus, differences in phylogenetic diversity could be attributable to genetic and familiar risks. The implications of the HLA-DR and DQ genes for microbiota alterations are well known. In Spanish children with relatives who had CD, the HLA-DQ2 haplotype was associated with a higher abundance of Firmicutes and Proteobacteria; but a lower proportion of Actinobacteria related to *Bifidobacterium* species, compared to children with low genetic risk [22]. Also, HLA-DQ2 and DQ8 were associated with higher abundance of *Bacteroides* and *Enterococcus* in infants at risk for CD from the USA and Italy [14]. Meanwhile, in Sweden, children with T1D at high genetic risk, were associated with differences in beta diversity and a high abundance of *Agathobacter*, *Blautia*, and *Dorea* genera [23]. The mechanisms by which HLA could modulate gut microbiota are related to class II molecules in peptide or bacterial polysaccharide presentation to T lymphocytes [24].

The main microbiota marker found in Group 2 was a higher abundance of *Agathobacter*. This genus has also been found to be more prevalent in children at high genetic risk for T1D [23]. A higher abundance of the *Agathobacter* genus and the Lachnospiraceae family, could be related to an increased risk of T1D and CD development. Xu et al. [25], published a two-sample Mendelian randomization data analysis finding a causal relationship between the higher abundance of the *Bifidobacterium* genus and T1D and CD development. In the present and previous studies in children, we did not find any important abundance of the Actinobacteria phylum (≤1%) or any *Bifidobacterium* genus.

Genetic and familiar risk was the major factor influencing the microbiota in our study. We found five taxa differentiators between study groups: Lachnospiraceae, Oscillospiraceae UCG-002, *Parabacteroides*, *Akkermansia*, and *Alistipes*. The abundance of Lachnospiraceae were higher in our children of Groups 1 and 2 than in those of Group 3; according to Krych et al. [26], this family correlated negatively with splenic FoxP3 + CD4+ Treg cells and with the delay of disease onset. More interesting, Lachnospiraceae had high representation in the gut microbiota before the onset of CD [7] and at the onset of T1D [27], highlighting its involvement in the development of these disorders.

Lachnospiraceae were positively correlated with the sucrose degradation pathway in our analysis. Previously, Biassoni et al. [8] found that sugar metabolism is an abundant metabolic pathway in the microbiota of children at T1D onset. When high concentrations of free sugars are consumed, the capacity for carbohydrate absorption in the small intestine can be surpassed, leading to the sugars reaching the colon [28]. This creates microenvironments that favor the overgrowth of certain bacteria, depending on the sugar type and bacterial metabolic needs, thereby altering gut microbiota diversity and profile [29]. In our study, the three groups had similar carbohydrate consumption, but we did not evaluate free sugars intake. However, it is well known that in Mexico children are the principal consumers of ultra-processed foods, with high free sugar content [30]. Hence, it is possible that overconsumption of free sugars, and genetic risk, is skewing microbiota towards a high abundance of saccharolytic bacteria such as Lachnospiraceae and *Agathobacter*.

On the other hand, *Alistipes* and *Akkermansia* were more abundant in our control children (Group 3) than the other two groups. *Alistipes* genus produces succinate, acetate, and propionate, and their protective role against diseases or participation in inflammatory processes is still being discussed [31]. However, *Alistipes* has been described as a strong factor that differentiates between healthy children and those who develop T1D before the disease onset [32]. In T1D patients at onset, GADA levels negatively correlate with *Alistipes* abundance, suggesting it as a predictive biomarker for T1D [27]. Additionally, *Alistipes onderdonkii* and *A. shanhii* were increased in healthy children’s fecal and duodenal microbiota, respectively compared to CD patients at onset [33]. Regarding *Akkermansia*, it has been found to be more abundant in healthy controls than in T1D patients at onset and their siblings [27], as well as in children with CD at onset [34]. Its role in gut health has been extensively studied. In particular, *Akkermansia muciniphila* is a symbiont that can use mucin as a carbon and nitrogen source, and has been associated with optimal intestinal barrier function and an adequate immune response [35].

The loss of intestinal barrier integrity is a crucial process in the development of CD. Although less directly related, the intestinal origin of T1D has been proven. The gastrointestinal tract represents the primary route for the entrance of diabetogenic antigens into the body’s tissues. Increased intestinal paracellular permeability has been found in T1D patients, even in the pre-onset stage [36]. In non-diabetic obese mice before T1D onset, there are lower levels of secretory IgA, decreased intestinal mucus production, diminished numbers of goblet cells, and altered profiles of intraepithelial lymphocytes. This impaired barrier allows bacterial translocation from the gut to the pancreatic lymph nodes, which could contribute to triggering T1D onset [37]. This disturbance can be induced by intraepithelial parasitic infections which also alter the gut microbiota. Previously we found a lower abundance of Ruminococaceae and Verrucomicrobioceae families and the *Akkermansia* genus in children with autoantibodies for T1D or CD [4].

Oscillospiraceae UCG_002 and *Parabacteroides* were more abundant in our Group 3 and were a protective factor against the presence of autoantibodies. This is consistent with the findings of Biassoni et al. [8], who reported a negative correlation between Oscillospiraceae and IAA in children with T1D at onset. Additionally, a longitudinal study conducted by Leonard et al. [7] reported lower abundance of Oscillospiraceae before to the onset of CD in children who developed the disease. The Oscillospiraceae family, also known as Ruminococcaceae, is composed of bacteria that can degrade the protein backbone of mucin [38]. They can selectively ferment glycans and dietary fibers, producing butyrate [39]. As we described earlier, butyrate play an important role in maintaining gut epithelial health. Yuan et al. [5] studied children at onset of T1D and in their elegant multi-omics and animal study, found that dysbiosis associated with this disease is characterized by increased LPS biosynthesis and decreased production of butyrate and bile acid metabolism, with destructive and protection effects, respectively.

Implications of *Parabacteroides* in human health is still controversial. Anti-inflammatory effects have been attributed to *P. distasonis* due to its production of acetate and decreased abundance of this bacteria have been related to disease [40]. However, more recently, a peptide from *P. distasonis* has been described to cause cross-reactivity, increasing CD8+ T cells while decreasing FoxP3+ Treg cells, accelerating T1D development in non-obese diabetic mice [41]. Still, participation of *Parabacteroides* in T1D and CD pathogenesis is unclear. It has increased abundance in children before to CD diagnosis [7], but it correlated positively with blood pH in children at T1D onset [8]. Thus, more investigation is needed to support the protective function of *Parabacteroides* found in our study.

*Alistipes*, *Akkermansia*, Oscillospiraceae UCG-002, and *Parabacteroides*, which were higher in our Group 3 were positively associated with at least one predicted pathway for amino acid biosynthesis. This agrees with the finding of Leonard et al. [14], who described a decreased abundance of amino acids metabolism pathways in infants with high genetic risk for CD. According to Yuan et al. [5], amino acid biosynthesis was more abundant in healthy children than in those with T1D at diagnosis. Additionally, Leonard et al. [7] found that a decreased abundance of functional pathways related to amino acid metabolism in children was associated with a high risk for CD. Bacterial amino acid production contributes to the host’s pool, which can be used in protein biosynthesis and energy acquisition. Furthermore, amino acids can be fermented into short-chain fatty acids that contribute to epithelial integrity and energy harvesting by colonocytes. For instance, glycine, threonine, glutamate, lysine, ornithine, and aspartate can be fermented into acetate, while threonine, glutamate, and lysine fermentation can lead to the production of butyrate [42].

The causative role of microbiota in CD and T1D development was proven in murine models, although the results are not straightforwardly translatable to the human context. Thus, longitudinal studies of genetically predisposed children followed from the birth have been key in clarifying the causality of microbiota alteration in CD and T1D development [7,9]. Our cross-sectional study contributes to this purpose. We demonstrate that genetic and familial risks, per se, influence microbiota markers that could increase the risk of developing autoimmunity. Furthermore, the absence of these risk factors is related to a protective effect of the microbiota against the presence of autoantibodies.

We consider a strength of our study to have a homogeneous population, but at the same time, this could also be a limitation. Since we focused on Mexican mestizo children in Northwest Mexico, it is important to take into account the specific characteristics of this population alongside our conclusions.

## 5. Conclusions

Our study provides evidence that the gut microbiota composition in children predisposed to CD and T1D depends on the genetic and familial risk. Our findings suggest that HLA-DQ2 and DQ8 influence the decrease in phylogenetic diversity, which may lead to lower stability and resilience of the gut microbiota. Furthermore, our results indicate that *Alistipes*, *Akkermansia*, Oscillospiraceae UCG-002, and *Parabacteroides* may play a protective role against the development of autoantibodies. Meanwhile, *Agathobacter* and Lachnospiraceae could be markers of increased risk, which may contribute to the development of autoimmunity. However, further research is needed to fully elucidate the mechanisms underlying the role of these bacterial families and genera in the development of autoimmunity for CD or T1D in different geographical and cultural regions.

We designed a cross-sectional study due to the challenges associated with conducting a longitudinal study in our context, where people are not used to going to the doctor for follow-up but only if they are ill. This approach enabled us to identify children at risk of CD or T1D and those who developed autoantibodies prior to the disease’s onset without the need for follow-up from birth. By reducing time and costs, this methodology may facilitate further research in this field and contribute to a better understanding of the causal role of the microbiota in the development of CD and T1D.

## Figures and Tables

**Table 1:** Characteristics and diet of children in the three study groups.

Variable	Group 1	Group 2	Group 3	*p*-Value
	(*n* = 18)	(*n* = 18)	(*n* = 17)	
Sex, female, %	72.2	66.7	64.7	0.884
Age, mean (SE), years	9.28 (0.39)	9.56 (0.42)	9.59 (0.30)	0.813
Weight, median (IQR), kg	33.70 (26.14–41.15)	32.45 (24.97–42.40)	36.65 (31.03–48.15)	0.299
Height, median (IQR), cm	1.37 (1.28–1.49)	1.39 (1.31–1.47)	1.37 (1.30–1.48)	0.969
BMI/age, mean (SE), Z-Score	0.69 (1.43)	0.64 (1.53)	1.43 (1.13)	0.173
**Diet**				
Total energy intake, mean (SE), kcal	1715.25 (61.84)	1675.17 (119.71)	1533.31 (76.35)	0.327
Carbohydrates intake, mean (SE), % *	54.80 (2.16)	56.82 (1.98)	53.31 (2.31)	0.517
Protein intake, mean (SE), % *	11.82 (0.84)	11.45 (0.79)	11.64 (0.67)	0.058
Fat intake, mean (SE), % *	33.98 (1.97)	33.63 (1.62)	36.59 (1.86)	0.466
Saturated fat intake, mean (SE), % *	10.46 (0.59)	9.65 (0.63)	11.84 (1.01)	0.130
Fiber intake, median (IQR), g/day	16.96 (12.60–20.74)	15.83 (10.24–24.34)	11.72 (9.16–19.18)	0.193

Group 1: children with genetic risk, without autoantibodies. Group 2: children with genetic risk, with autoantibodies. Group 3: children without genetic risk. IQR: interquartile rank (Q1–Q3); SE: standard error; BMI: body mass index. * Percentage of the total energy intake.

## Data Availability

The data presented in this study are available upon request from the corresponding author.
